# Uncovering Unique Effects in Preclinical Alzheimer's Disease with Digital Cognition

**DOI:** 10.1002/alz70857_099582

**Published:** 2025-12-24

**Authors:** Jason J. Hassenstab, Matthew S. Welhaf

**Affiliations:** ^1^ Washington University in St. Louis, St. Louis, MO, USA; ^2^ Knight Alzheimer Disease Research Center, St. Louis, MO, USA

## Abstract

By definition, there are no overt cognitive changes in the preclinical stages of Alzheimer's disease (AD). Technologies for cognitive assessments have evolved to such a degree that they are more than digital substitutes for conventional tests and can reveal unique aspects of cognitive changes in AD prior to symptom onset. In this *Perspectives* session on preclinical AD, we will present on recent work from our lab and from others highlighting how digital approaches can uncover signal in preclinical AD that conventional cognitive testing cannot.

Using remote methods to distribute cognitive assessments across multiple testing sessions provides tremendously rich data to test hypotheses about how AD pathology impacts a multitude of cognitive functions. While one‐off, in‐clinic assessments can provide a snapshot of cognitive status, constructs like within‐ and between‐person variability, learning effects, forgetting rates, time of day effects, and short‐term effects of stress and sleep can be modeled with high‐frequency digital cognitive approaches that move assessments away from the clinic and into the daily lives of research and clinical populations.

In sporadic AD, we have shown that older adults are highly capable of participating in remote digital studies^1^ and produce valid and reliable estimates of cognitive change that correlate well with in‐clinic tests and AD biomarkers^2^. Because tests are repeatedly administered, we can model variability over different timescales, and we have shown that in participants with genetic risk for AD or with elevated AD biomarkers, cognition varies more from day‐to‐day^3^, between evening and morning hours^4^, and even from moment‐to‐moment^5^ than those at less genetic risk and with normal AD biomarker levels. Recent work using technology to measure learning and also forgetting rates in preclinical AD have shown reduced learning over several consecutive days in biomarker‐positive participants^6,7^ and increased forgetting rates in individuals at genetic risk for AD^8^. Finally, we will present recent work from our laboratory examining effects of momentary changes in mood and sleep on cognition in preclinical AD.

Digital cognitive assessments demonstrate sensitivity in preclinical AD and can capture clinically meaningful constructs that could be used as novel outcomes in prevention trials.

